# A scientometric analysis of research trends on targeting mTOR in breast cancer from 2012 to 2022

**DOI:** 10.3389/fonc.2023.1167154

**Published:** 2023-08-10

**Authors:** Xizhou Zhang, Jinyao Wu, Qiuping Yang, Huiting Tian, Lingzhi Chen, Daitian Zheng, Zeqi Ji, Jiehui Cai, Yexi Chen, Zhiyang Li

**Affiliations:** Department of Thyroid, Breast and Hernia Surgery, General Surgery, The Second Affiliated Hospital of Shantou University Medical College, Shantou, Guangdong, China

**Keywords:** breast cancer, mTOR, scientometrics, WoS, bibliometrix, VOSviewer

## Abstract

Over the past decade, thousands of articles have been published on the mechanistic target of rapamycin (mTOR) and its role in breast cancer. However, the variability and heterogeneity of academic data may impact the acquisition of published research information. Due to the large number, heterogeneity, and varying quality of publications related to mTOR and breast cancer, sorting out the present state of the research in this area is critical for both researchers and clinicians. Therefore, scientometric techniques and visualization tools were employed to analyze the large number of bibliographic metadata related to the research area of mTOR and breast cancer. The features of relevant publications were searched from 2012 to 2022 to evaluate the present status of research and the evolution of research hotspots in this particular field. Web of Science was utilized to extract all relevant publications from 2012 to 2022. Subsequently, Biblioshiny and VOSviewer were utilized to obtain data on the most productive countries, authors, and institutions, annual publications and citations, the most influential journals and articles, and the most frequently occurring keywords. In total, 1,471 publications were retrieved, comprising 1,167 original articles and 304 reviews. There was a significant rise in publications between 2015 and 2018, followed by a sharp decline in 2019 and a rebound since then. The publication with the highest number of citations was a 2012 review authored by Baselga et al. The United States had the highest number of publications, citations and connections among all countries. Oncotarget had the highest number of published articles among all the journals, and José Baselga had the strongest links with other authors. Excluding the search topics, the most frequently used words were “expression” (n = 297), “growth” (n = 228), “activation” (n = 223), “pathway” (n = 205), and “apoptosis” (n = 195). mTOR is crucially involved in breast cancer pathogenesis, but its exact mechanism of action remains controversial and warrants further investigation. The scientometric analysis provides a distinct overview of the existing state of research and highlights the topical issues that deserve further exploration.

## 1 Introduction

A 2012 study showed that breast cancer is the second most frequently diagnosed cancer globally and the predominant cancer among femals (1, 2). In 2012, approximately 1.67 million new cases of cancer were reported worldwide, being the fifth most common cause of cancer death (522,000 deaths) (1). In 2020, breast cancer has officially become the most commonly diagnosed malignancy in the world, as well as the second major cause of cancer mortality after lung cancer (3–5). In China, breast cancer now ranks as the most commonly diagnosed cancer in females, accountings for 12.2% of all new breast cancer diagnoses worldwide and 9.6% of breast cancer deaths around the world (6). Breast cancer has become a worldwide public health problem due to its complex etiology and the adverse effects of treatment (2, 7). Considering the increasing prevalence of breast cancer, there is an imperative to develop an accurate and effective treatment to overcome this pervasive global health issue.

The mechanistic target of rapamycin (mTOR) is a member of the phosphatidylinositol 3-kinase-related kinase (PIKK) family and is found in many mammalian cells (8, 9). The mTOR signaling pathway regulates multiple major cellular life processes and is also involved in many pathological conditions, including cancer (8). The mTOR signaling pathway’s oncogenic activation contributes to the growth, proliferation, and survival of cancer cells, emphasizing the potential of mTOR pathway inhibitors to serve as efficient anti-cancer drugs (9, 10). Many studies have shown that mTOR inhibitors can also be effectively applied role in breast cancer treatment (11–15). For example, the mTOR inhibitor everolimus has shown anticancer activity when combined with exemestane in the treatment of advanced breast cancer (15). Some mechanisms of the mTOR signaling pathway in the occurrence and development of cancer have been studied to some extent (9, 16). However, the specific mechanism of mTOR in the occurrence and development of breast cancer is still unclear and requires further study.

Nevertheless, no scientometric analysis of mTOR and breast cancer has been conducted in recent years. The bibliometric analysis of mTOR and breast cancer promotes a better understanding of the frontier knowledge and research hotspots in this field. Therefore, a scientometric analysis was conducted on articles related to mTOR in breast cancer to outline the current state of research. This analysis aimed to obtain annual publication and citation date, top-producing countries and authors, influential journals, and keyword analysis for the past decade in the relevant field to provide researchers with intriguing directions for study.

## 2 Methods

### 2.1 Data collection

Recently, multiple publications have investigated the role of the mammalian target of rapamycin (mTOR) pathway in breast cancer. To ensure the quality of the collected data, the Web of Science (WoS) core collection database was used, which included Science Citation Index Expanded (SCI-EXPANDED) (2003–present), Social Sciences Citation Index (SSCI) (2003–present), Emerging Sources Citation Index (ESCI) (2018–present), Index Chemicus (IC) (1993–present), and Current Chemical Reactions (CCR-EXPANDED) (1985-present). The following terms were used for the search: #1, TS=(“mechanistic target of rapamycin”) OR (“mammalian target of rapamycin”) OR (“mTORC1 OR mTORC2”); #2, TS=(“Breast Cancer”) OR (“Breast Neoplasm”); #3, “#1” AND “#2”. The data analysis time was set to January 17th, 2023. From the Web of Science Core Collection database, 1,559 publications were retrieved between 2012 and 2022. Then the document type was restricted to article or review, and the language type was set to English only. Thereafter, 1,471 publications were screented, including 1,167 articles (79.49%) and 304 review articles (20.51%). A total of 88 publications were excluded from the analysis: 8 non-English publications, 37 meeting abstracts, 11 editorial materials, 12 early access, 5 book chapters, 7 proceedings papers, 3 corrections, 1 retraction, 2 retracted publications, and 2 letters.

### 2.2 Data analysis

Preliminary application of the online “Analyze Results” feature of the Web of Science (WoS) yielded information on the year of publications, type of documents, publishers, affiliations, research areas, languages, authors, funding agencies, countries, journals, and open access for these publications. Additional information, such as the number of articles citing and citing articles not self-cited, the sum of the cited times and not self-cited counts, the average number of citations per term (ACI), and the h-index, is also obtained from the “Create Citation Report” feature of WoS.

Biblioshiny (Bibliometrix’s web interface) and VOSviewer were used to analyze the collected data.

Bibliometrix (version 4.1.0) is an open-source tool that can perform a scientometric analysis of data collected from WoS (17). The raw data of the dataset were imported from WoS into the Biblioshiny website to obtain the main information of the articles, including the number of sources, the time span, the type of documents, the number of documents, the content of the documents, the number of references, and the collaboration between authors. The obtained information was screened to meet the inclusion criteria. In addition, the annual scientific results, authors (top contributing authors, author impact ranks by h-index and m-index), sources (most contributing journals and source dynamics), national and affiliate contributions, and high-frequency keywords in this research area were analyzed. A co-occurrence network for the “keywords plus” can be used to help detect research hotspots. The relationship between the best authors, the most productive affiliates, and the highest contributing countries was summarized through a three-field plot.

VOSviewer (version 1.6.17) was used as a tool for network analysis in order to visualize and analyze the top cited documents, constructing a reference co-citation network analysis and a keyword co-occurrence network analysis (18). Furthermore, the strength of the links between the different affiliations, countries and authors in our study was analyzed using this software.

**Figure 1:**
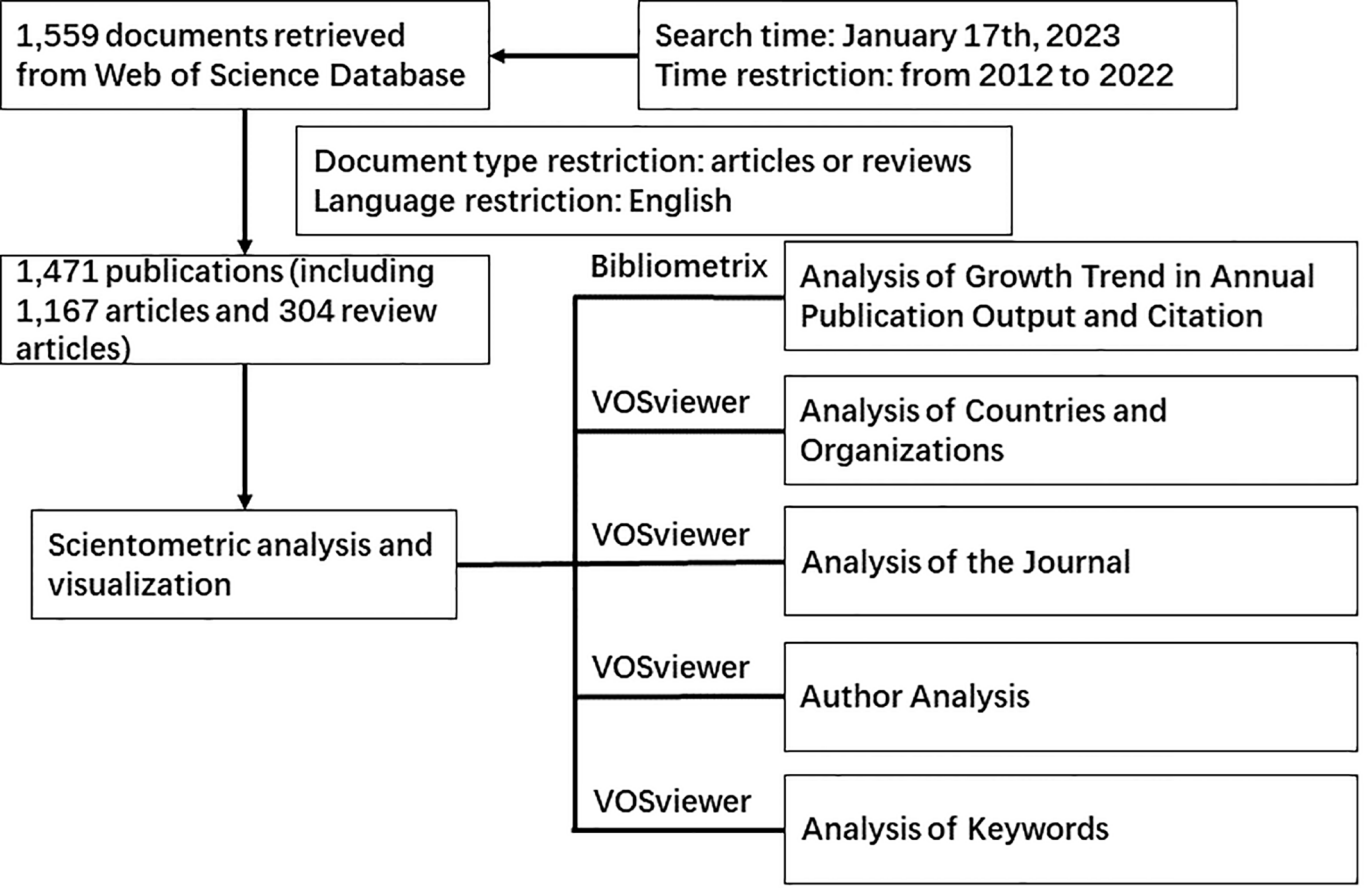
Flowchart of data collection and analysis.

**Figure 1** presents the document collection steps and the analysis results. No ethical committee approval was required for this study.

## 3 Results

### 3.1 Analysis of growth trend in annual publication output and citation

In total, 1,471 publications were retrieved from the WoS core collection database using the above methodology and country restriction.

**Figure 2:**
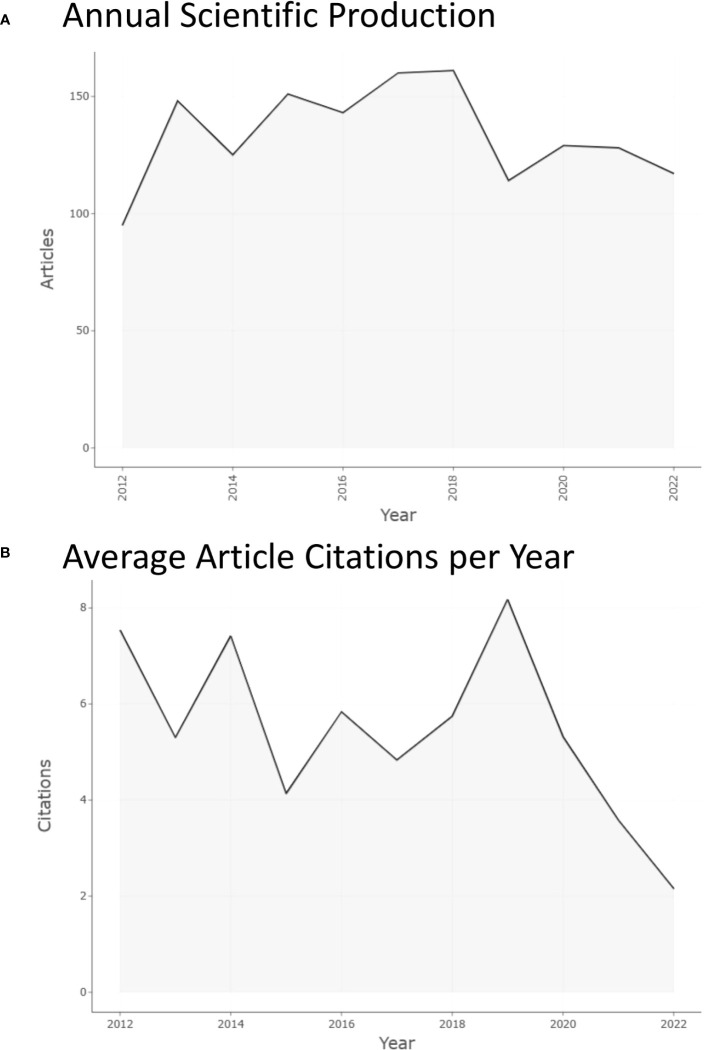
Annual scientific production and average article citations per year from 2012 to 2022 in this research field. **(A)** Annual scientific production from 2012 to 2022 in this research field. **(B)** Average article citations per year from 2012 to 2022 in this research field.

An upward trend in the number of publications was observed from 95 articles published in 2012 (6.46%) to 161 articles published in 2018 (10.94%), as displayed in **Figure 2A**. Notably, the number of articles published in 2019 suddenly dropped to 114 (7.75%). Subsequently, the number of articles published continued to rise and 129 publications were published in 2020 (8.77%), and then decreased to 117 publications in 2022 (7.95%). Between 2012 and 2022, the highest growth rate in the number of articles published (55.79%) was seen in 2013 while the lowest growth rate (-29.19%) was in 2019. 2018 was the year with the largest contribution of 161 publications, and 2012 was the year with the lowest contribution of 95 publications. One of the criteria used to assess an article’s quality is citation analysis (19). An article’s scientific impact can be inferred from how frequently it is cited in other papers (19). **Figure 2B** shows the average number of citations per year from 2012 to 2022, with the largest number in 2019 (8.2), a tortuous fluctuation from 2012 to 2019, and a rapid decline from 2019 to 2022.

**Figure 3:**
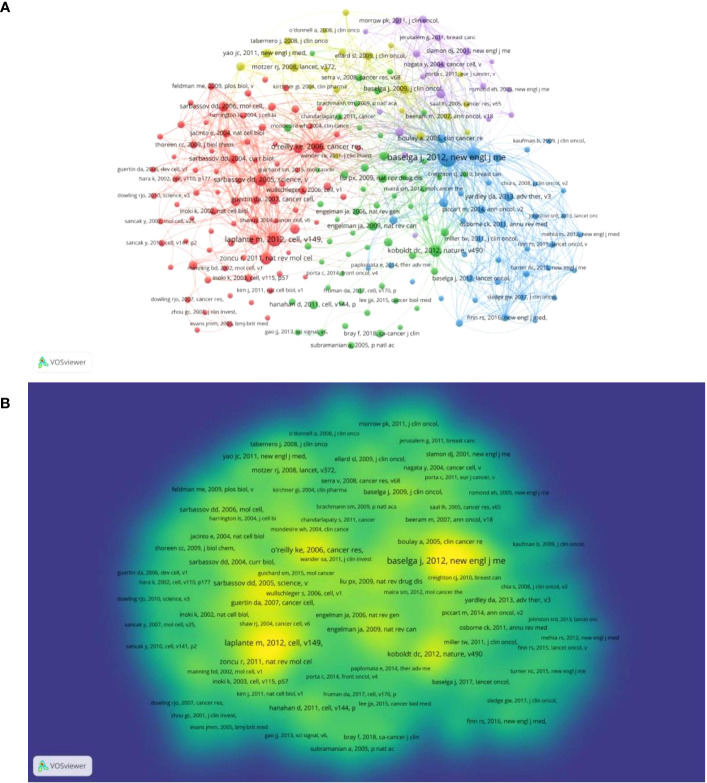
Co-cited network mapping of cited references of these documents. **(A)** Network map of co-citation between references with more than 20 citations. **(B)** Density map of co-citation between references with more than 20 citations.

**Figure 3** depicts a co-cited network diagram of the publication references by using VOSviewer. In total, 213 references met the requirement, with a minimum number of citations of 20 for a cited reference. A higher number of references was illustrated by a larger node (**Figure 3A**). Greater yellowness indicated a higher number of co-citations (**Figure 3B**). The color of one item showed the cluster it belongs to, based on the amount of content in the clusters, from highest to lowest in order of red, green, blue, yellow, and purple. In **Figure 3A**, the mentioned references were grouped into five groups, the top cluster with 80 items indicated the most intriguing research area, shown in red. The rest of the clusters were ranked in order of green (56 items), blue (39), yellow (20) and purple (18). In **Figure 3**, the publication published by Baselga (2012) got the most citations (240 citations), indicating that this reference was highly valued (11). The five references Baselga J (2012) (240 citations) (11), Laplante M (2012) (150 citations) (8), O’Reilly Ke (2006) (120 citations) (20), Bachelot T (2012) (119 citations) (12), and Zoncu R (2011) (111 citations) (16) received the most citations (**Table 1**). Laplante M and Zoncu R’s studies reported some of the known mechanisms of action of the mTOR signaling pathway (8, 16). They mentioned that mTOR is a protein kinase that senses energy, nutrient, growth factor and stress signals inside and outside the cell and regulates cell growth, metabolism, autophagy and aging processes through two complexes (mTOR complexes 1 and 2). Their exploration of the mTOR mechanism played a seminal role in the field and therefore received high citations. Baselga J, O’Reilly Ke, and Bachelot T’s studies demonstrate the value of mTOR inhibitors in the treatment of breast cancer (11, 12, 20). Additionally, their research provided a theoretical basis for the development of more effective combination therapies in clinical settings. These highly cited articles not only demonstrate the important role of the mTOR signaling pathway in the development of breast cancer, but also provided a theoretical rationale and clinical evidence for mTOR inhibitors as a potential therapeutic strategy. Moreover, the identification of highly cited articles and their contents can inform the development of new research directions and therapeutic strategies in the field of breast cancer.

**Table 1:** Top 5 cited articles contributing to this research area.

Article	Main research content
Baselga J, 2012, N Engl J Med	This article mainly studied the efficacy and safety of everolimus and exemestane combination therapy for patients with hormone-receptor-positive advanced breast cancer. Everolimus is a drug that inhibits the mTOR signaling pathway, which is related to endocrine resistance. The authors randomly assigned 724 patients with hormone-receptor-positive advanced breast cancer who had received previous nonsteroidal aromatase inhibitor therapy, to receive everolimus and exemestane combination therapy or exemestane and placebo combination therapy. The results showed that everolimus and exemestane combination therapy significantly prolonged progression-free survival, but also accompanied by more adverse events.
Laplante M, 2012, Cell	This article mainly studies the structure, function, and regulation mechanisms of the mTOR signaling pathway, and its role in cell growth, metabolism, autophagy, aging and disease. The article also introduces some pharmacological intervention strategies targeting the mTOR signaling pathway, and their applications and limitations in the clinic.
O’Reilly Ke, 2006, Cancer Res	This article mainly studies the effect of mTOR inhibitors on Akt signaling pathway in tumor cells. The authors found that mTOR inhibitors can activate Akt by up-regulating IRS-1 protein levels and relieving negative feedback inhibition of IGF-I receptor signaling. This Akt activation may attenuate the antitumor effects of mTOR inhibitors, while combination therapy with IGF-I receptor inhibitors and mTOR inhibitors may enhance cell cycle arrest and apoptosis induction. The authors validated this hypothesis *in vitro* and *in vivo* models and provided a theoretical basis for developing more effective combination therapies in clinical settings.
Bachelot T, 2012, J Clin Oncol	This article mainly studied the efficacy and safety of everolimus combined with tamoxifen for patients with metastatic breast cancer resistant to aromatase inhibitors (AI). They hypothesized that inhibition of the mTOR signaling pathway could restore sensitivity to endocrine therapy, especially in patients with AI resistance. They used an open-label, randomized, phase II trial design to divide patients into two groups, one receiving tamoxifen monotherapy and the other receiving tamoxifen plus everolimus combination therapy. They found that the combination therapy group had significantly higher clinical benefit rate, progression-free survival, and overall survival at 6 months than the monotherapy group, and adverse reactions were tolerable and manageable.
Zoncu R, 2011, Nat Rev Mol Cell Biol	This article mainly studies the role and regulation of the mTOR signaling pathway in cell growth, metabolism, and aging. mTOR is a protein kinase that can sense energy, nutrients, and stress signals, and it can form two different complexes, namely mTORC1 and mTORC2. These two complexes have different substrates and functions, and are also influenced by different upstream factors. The authors review the molecular composition, regulatory inputs, and cellular outputs of the mTOR signaling pathway, and discuss how to use this knowledge to treat cancer, diabetes, and delay aging.

Although a high number of citations can indicate a significant contribution to a research field, other factors may influence the number of citations, such as the popularity of the research topic or the impact factor of the journal. Therefore, the citation analysis results were subjected to further analysis.

### 3.2 Analysis of countries and organizations

**Figure 4:**
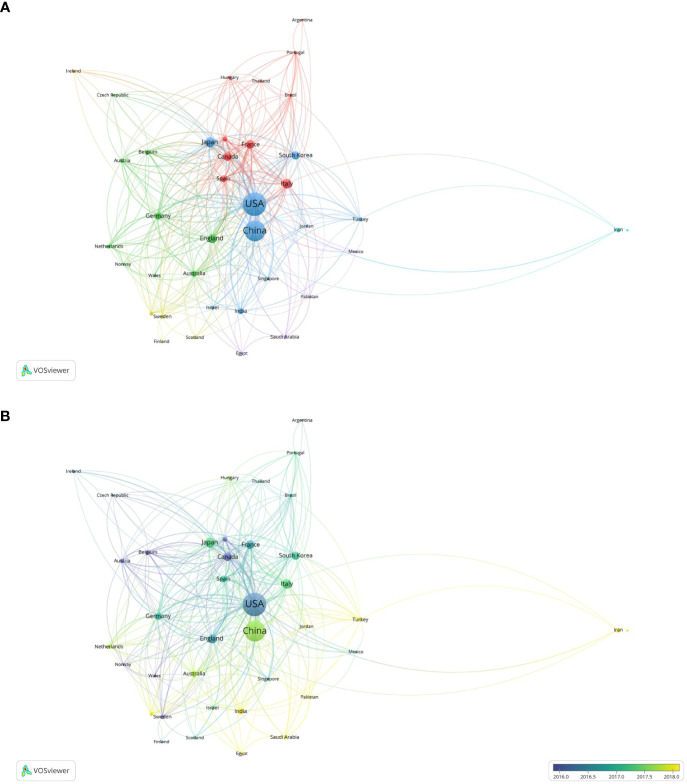
Co-authorship analysis of countries in this field. **(A)** Network map of co-authorship between countries with more than five publications. **(B)** Overlay map of co-authorship between countries with more than five publications. (The blue color represents the earlier years; the yellow color represents the more recent years.).

The 1,471 retrieved documents originated from 62 countries and regions. The most productive nation in terms of number of published papers was the United States (n = 433, accounting for 29.4%), followed by China (n = 396, 26.9%), Japan (n = 64, 4.4%), the United Kingdom (n = 63, 4.3%), and Korea (n = 62, 4.2%). **Table 2** shows the top ten referenced nations. The publications from the United States (n = 21,632) received the most citations, followed by China (n = 10,162), Canada (n =2,247), France (n = 1,986), and Italy (n = 1,973). In co-authorship analysis, the minimum number of files from a country was set to 5, and a total of 39 countries met the threshold. Total link strength refers to the total number of co-authored works between the target country/region and other countries/regions (21). It is defined as the number of other keywords that the specified keyword co-occurs with in the review database. The top five countries in terms of total link strength were the United States (total link strength = 411), China (total link strength = 144), France (total link strength = 134), England (total link strength = 121), and Italy (total link strength = 106), as displayed in **Figure 4A**. The diameter of the circle represents the overall strength of the links between the various nations. According to the frequency of their co-occurrences, the distance between circles indicates the intensity of their link. With time, certain developing countries, such as China (total link strength = 144), India (total link strength = 44), Turkey (total link strength = 30), Egypt (total link strength = 14), and Jordan (total link strength = 11), made significant contributions in this area (**Figure 4B**).

**Table 2:** Top 10 cited countries contributing to this research area.

Country	Total production	Total citations	Average article citations
USA	433	21,632	49.96
China	396	10,162	25.66
Japan	64	1,101	17.20
United Kingdom	63	1,944	30.86
Korea	62	1,037	16.73
Italy	61	1,973	32.34
Canada	55	2,247	40.85
France	48	1,986	41.38
Germany	34	888	26.12
India	28	721	25.75

Total citations: Total citations are the total number of times a journal was cited by all journals in the database during the JCR year; Average article citations: The average number of article citations is the result of dividing the total number of citations by the total production.

Overall, 2,150 institutions participated in this field. The University of Texas MD Anderson Cancer Center (n =94, 6.4%), McGill University (n = 85, 5.8%), Memorial Sloan Kettering Cancer Center (n = 61, 4.1%), Taipei Medical University (n =60, 4.1%), and Sun Yat-Sen University (n =49, 3.3%) were the top five institutions in terms of contributing articles (**Table 3**). In addition, the minimum number of files from an organization was set to 5 for the co-authorship analysis, with 189 meeting the threshold. The University of Texas MD Anderson Cancer Center (total link strength = 102), Memorial Sloan Kettering Cancer Center (total link strength =98), the University of California-San Francisco (total link strength =77), the University of Toronto (total link strength =53), and Dana-Farber Cancer Institute (total link strength =52) ranked the highest in terms of total link strength (**Supplementary Figure 1**).

**Table 3:** Top 15 productive institutions promoting this field of study.

Organization	Total production	Total citations	Total link strength
The University of Texas MD Anderson Cancer Center	94	3,996	102
McGill University	85	936	20
Memorial Sloan Kettering Cancer Center	61	2,100	98
Taipei Medical University	60	303	13
Sun Yat-Sen University	49	940	23
Zhejiang University	44	736	16
Nanjing Medical University	43	594	15
University of Michigan	43	1,087	21
Fudan University	36	779	20
Harvard Medical School	36	650	38
Shanghai Jiao Tong University	36	1,153	18
The University of Toronto	35	2,981	53
University of California, San Francisco	34	3,675	77
Washington University	32	448	15
China Medical University	31	454	11

### 3.3 Analysis of the journal

These 1,471 documents appeared in 466 different journals. **Table 4** lists the top ten most influential journals in this field. The top 10 journals published 291 articles, accounting for 19.78% of the total number of articles published. Oncotarget had the most documents published (n = 61) and was followed by Oncology Letters (n =34), International Journal of Molecular Sciences (n =32), Oncogene (n = 32), and PLOS One (n = 31). The Hirsch index, sometimes known as the h-index, is widely considered as a key metric of scientific contribution (22). Oncogene and Oncotarget shared the highest h-index of 24 followed by Breast Cancer Research (h-index = 19), the International Journal of Molecular Sciences (h-index = 18), and PLOS One (h-index = 18). Oncotarget is a major journal in the field of oncology. but has not been published in the mTOR since 2018. PLOS One, Oncogene, International Journal of Molecular Sciences, and Oncology Letters are still active in this area, with the International Journal of Molecular Sciences showing a relatively stable publication growth rate. (**Supplementary Figure 2**).

**Table 4:** Source impact of the top 10 journals publishing in this field.

Source	h-index	m-index	TC	Articles	PY start
Oncogene	24	2.000	1,977	32	2012
Oncotarget	24	2.182	1,936	61	2013
Breast Cancer Research	19	1.583	987	29	2012
International Journal of Molecular Sciences	18	1.500	1,680	32	2012
PLOS One	18	1.636	1,064	31	2013
Breast Cancer Research and Treatment	17	1.417	988	29	2012
Oncology Letters	15	1.250	686	34	2012
Journal of Biological Chemistry	14	1.167	558	17	2012
Oncology Reports	13	1.182	432	19	2013
Cancer Treatment Reviews	12	1.000	1,026	12	2012

h-index, sometimes known as the Hirsch index, is widely considered as a key metric of scientific contribution; m-index, a correction of the h-index for time; TC, Total citations; PY, Publication year.

### 3.4 Author analysis

Data from 9,437 authors who contributed to the 1,471 publications were obtained, comprising 41 single-author documents (from 39 distinct authors) and 1,430 multi-author documents. Each document had an average of 8.04 co-authors. The most prolific author was Wang J, who contributed to 22 publications (1.50%), followed in order by Wang Y (n = 18, 1.22%), Zhang Y (n = 18, 1.22%), and Baselga J (n = 16, 1.09%) (**Table 5**). Only one author wrote more than 20 pieces, while fifteen authors have written more than 10. In **Supplementary Figure 3**, the dot size corresponds to the number of publications, and the color shade corresponds to the ratio of the overall count of citations every year. Baselga J was active in the first five years while Wang J was active in the last five years. In addition, Wang Y’s annual publication volume has experienced ups and downs over the past 10 years.

**Table 5:** Top 10 authors who have contributed to the study of mTOR and breast cancer.

Authors	Articles	Articles fractionalized	h-index	m-index	TC	PY_start
Wang J	22	2.46	14	1.167	1,124	2012
Wang Y	18	2.17	12	1.000	605	2012
Zhang Y	18	1.90	13	1.083	643	2012
Baselga J	16	1.29	15	1.250	3,052	2012
Li L	15	1.69	11	1.100	308	2014
Li Y	15	1.80	9	0.900	351	2014
Wang Q	15	1.92	11	1.222	942	2015
Liu Y	14	1.20	11	0.917	694	2012
Campone M	13	1.26	9	0.750	2,497	2012
Wang L	13	1.58	10	0.833	507	2012

h-index, sometimes known as the Hirsch index, is widely considered as a key metric of scientific contribution; m-index, a correction of the h-index for time; TC, Total citations; PY, Publication year.

The top 5 authors in terms of time cited were are Baselga J (n = 3,052), Bachelot T (n = 2,971), Rugo HS (n = 2,748), Burris HA (n = 2,549) and Campone M (n = 2,497). Baselga J ranked first in terms of the h-index (h-index = 15), followed by Wang J (h-index = 14), Zhang Y (h-index = 13), and Wang Y (h-index = 12). Nine of the most productive authors had an h-index greater than 10. The m-index is a correction of the h-index for time (22) and provide a useful scale to compare scientists of different seniority which can predict future achievements (22). Baselga J showed the highest m-index of 1.250 followed by Wang Q (m-index = 1.222), Wang J (m-index = 1.167), Li L (m-index = 1.100), and Zhang Y (m-index = 1.083).

**Figure 5:**
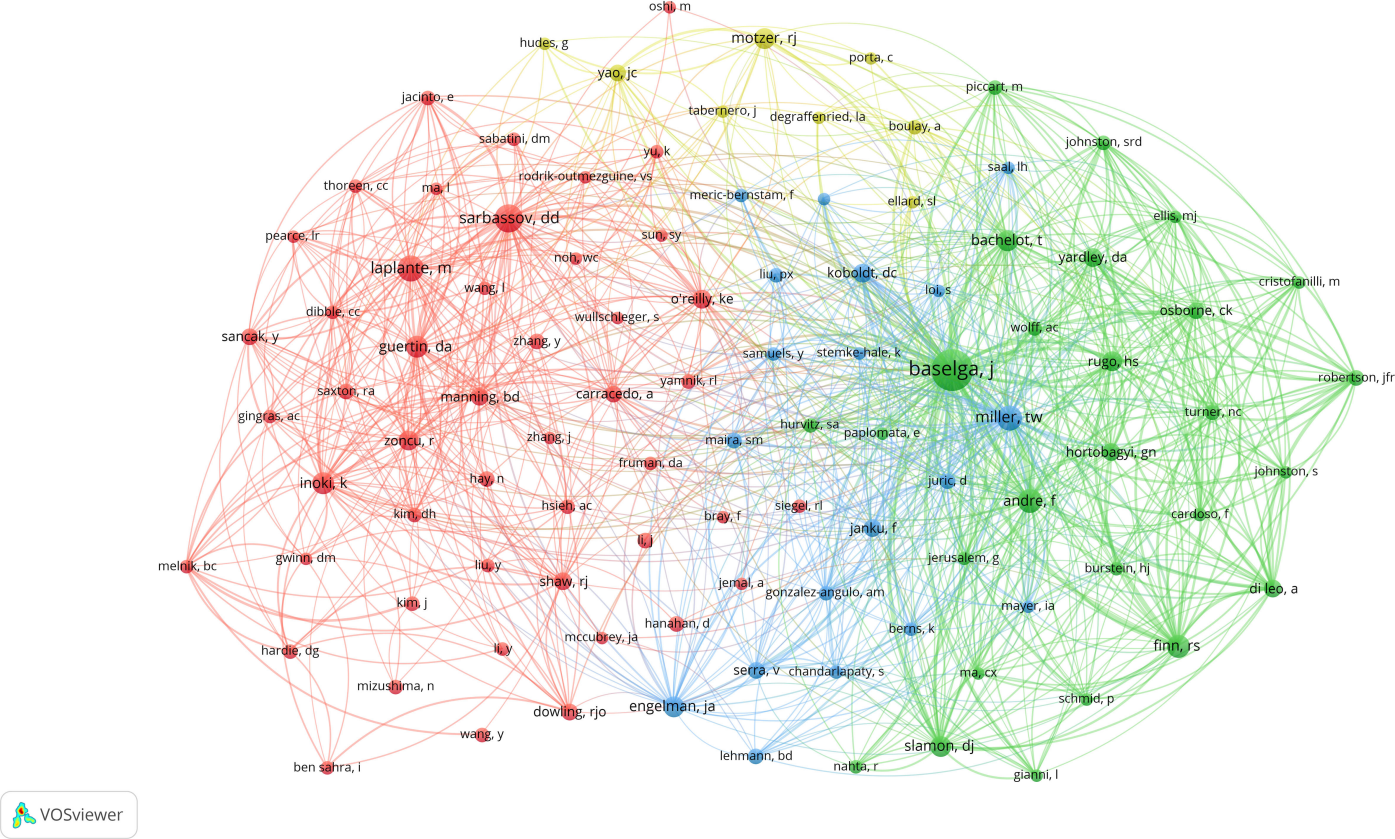
Network map of co-citations between authors with the minimum citations over 50 times.

To undertake author co-citation analysis, the VOSviewer program was employed (**Figure 5**). A total of 102 authors were examined, with a minimum of 50 citations. Baselga J (total link strength = 8,004), Miller TW (total link strength = 3,375), Andre F (total link strength = 2,875), Finn RS (total link strength = 2,721), and Sarbassov DD (total link strength = 2,677) were the top five authors in the co-citation analysis and had the strongest links.

The links between outstanding authors (left), the most productive nations (middle), and institutions (right) were displayed using a three-field plot (**Supplementary Figure 4**). The size of the nodes indicated their contribution, and the thickness of the lines indicates how closely they were connected. The United States has the most connections (n = 19), followed by China (n = 18) and Canada (n = 10). Taipei Medical University is the most significant contributor, followed by McGill University and the University of Texas MD Anderson Cancer Center. Campone M, Baselga J, and Liu Y were the top three authors with the most international collaboration. The institution with the most contact with China was Taipei Medical University, and the author with the most contact was Wang J. The most contacted institution in the United States was The University of Texas MD Anderson Cancer Center, and the most contacted author was Baselga J.

### 3.5 Analysis of keywords

**Figure 6:**
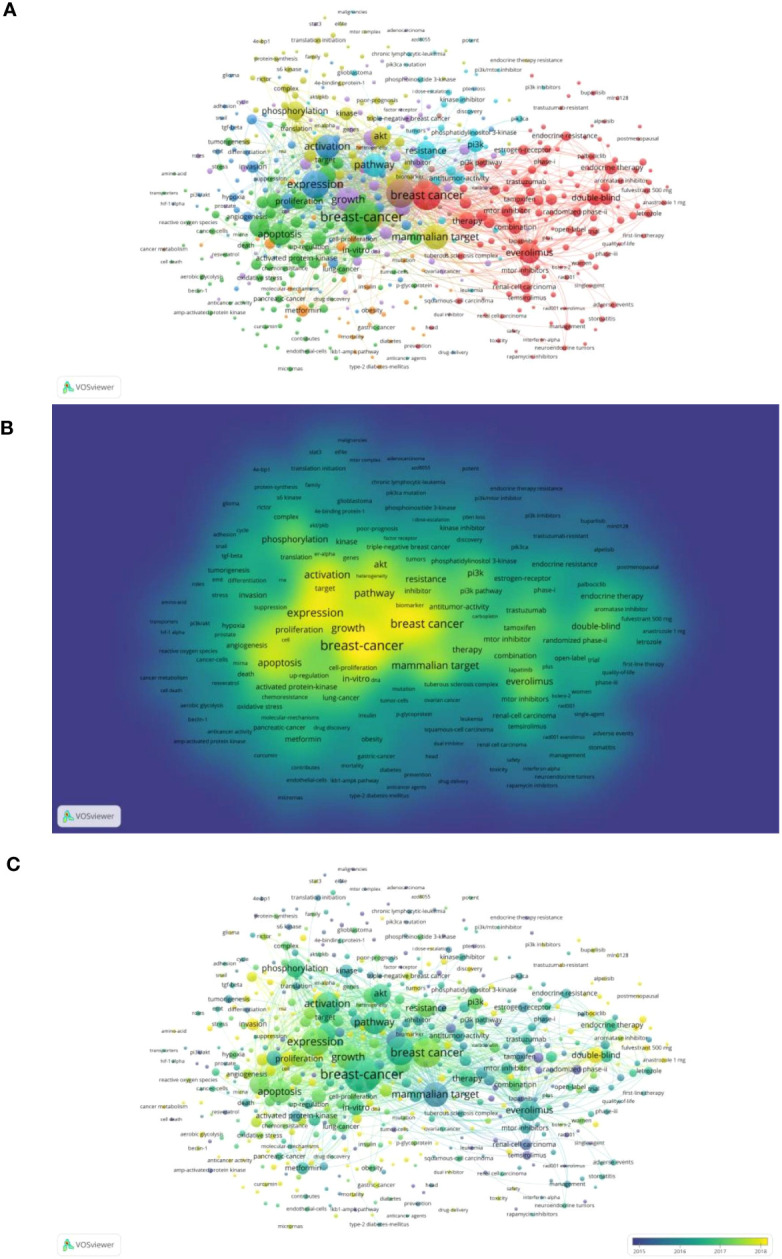
Co-occurrence analysis of keywords in this field. **(A)** Network map of keywords with more than 5 occurrences. **(B)** Density Visualization Map of keywords with more than 5 occurrences. **(C)** Overlay Visualization Map of keywords with more than 5 occurrences. (The blue color represents the earlier years; the yellow color represents the more recent years.).

VOSviewer was employed to analyze a total of 550 keywords that appeared with a frequency of over 5 instances. The entire co-occurrence network was separated into different clusters using VOSviewer’s clustering tool. The more relevant the keywords, the more likely they were to be placed in the same cluster and displayed in the same color. 8 clusters were formed during network visualization analysis of the chosen keywords (**Figure 6A**). Those will help us figure out where to focus our research in this area. The words “expression” (n = 297), “growth” (n = 228), “activation” (n = 223), “pathway” (n = 205), and “apoptosis” (n = 195) were frequently used (**Figure 6B**). In addition, the hue of the Overlay Visualization Map denotes the average time with which these keywords appear in research publications (**Figure 6C**). The later the keyword appeared, the more yellow it was displayed. Over time, the focus of the research field shifted from the application of mTOR inhibitors in the treatment of breast cancer to the mechanisms of the mTOR signaling pathway in the development of breast cancer, to the mechanism of resistance of mTOR inhibitors in the treatment of breast cancer, and the use of mTOR inhibitors in triple-negative breast cancer. Meanwhile, the keywords “cdk4”, “triple negative breast cancer”, “esr1 mutations” and “multicenter” appeared in recent years.

A thematic map was generated by using keyword-plus analysis. The words or phrases referred to as “keywords plus” were those that frequently appeared in the titles of references cited in an article but were absent from the article’s actual title (23). **Supplementary Figure 5** consists of four quadrants: (a) Motor themes (first quadrant) are well developed and vital for the research field, evidenced by their high centrality and density. (b) Highly developed and isolated themes (second quadrant) have a high density but a low centrality, indicating that they are now less important because they lack connections to other themes, including words “double-blind”, “postmenopausal women”, and “phase-II trial”. (c) Emerging or declining themes (third quadrant): they are low in centrality and density, these topics are less developed and less relevant in this field of research. (d) Basic themes (fourth quadrant): they are highly centered but low-density, they are important general themes in this field of research (23), including the words “mammalian target”, “therapy”, and “everolimus”. These keywords plus are grouped into three different colored clusters. As seen in **Supplementary Figure 5**, cluster 1’s (red color) keywords (“mammalian target”, “therapy”, and “everolimus”) were basic themes in this research field, while cluster 2’s (blue color) keywords (“double-blind”, “postmenopausal women”, and “phase-II trial”) were in the highly developed and isolated themes. Futhermore, cluster 3’ s (green color) keywords (“breast-cancer”, “expression”, “growth”) are in the center region of the graph, indicating that these keywords plus were highly associated in this region.

## 4 Discussion

Using Bibliometrix and VOSviewer, a scientometric analysis of the outputs of publications concerning mTOR and breast cancer was performed for the period 2012-2022. As of January 17th, 2023, the overall number of publications in this filed was 1,471. The number of publications showed a tortuous increase from 2015 and 2018, but suddenly plummeted in 2019 and has since rebounded, which indicates a renewed interest in this area of research. Four of the top 10 cited publications were published in 2012. Two of them focused on the use of the mTOR inhibitor everolimus in breast cancer treatment (11, 12), providing further evidence that everolimus can exhibit anticancer activity when added to endocrine therapy. A significant milestone was the paper written in 2012 by Baselga et al, which demonstrated the efficacy of the mTOR inhibitor everolimus in the treatment of breast cancer through a double-blind phase 3 study (11). An article published in the International Journal of Molecular Science reveals the molecular composition and regulation mechanism of mTOR, which is also one of the top 10 cited articles (24).

The global trend in medicine leaned toward international cooperation (25). In total, 62 countries and regions contributed to this field of research. The United States had the most publications, citations, and strength of connection with other countries. Among the 10 most contributing institutions in this research area, four were located in the United States, five in China, and the remaining one in Canada. Four of the top five institutions in terms of total link strength were located in the United States. The United States has made the highest contribution in this area of research and has the closest cooperation with other nations and institutions. Moreover, the increasing contribution of some developing countries to this field of research indicates the growing importance of this research area.

Among the 466 journals involved, the annual output of the journals International Journal of Molecular Sciences, Oncogene, Oncology Letters, and PLOS One in this field remained stable. In contrast, Oncotarget has not published in this field since 2018. Therefore, researchers with an interest in this field can focus on these journals at the time of article submission.

Wang J (n = 22, 1.50%), Wang Y (n = 18, 1.22%), and Zhang Y (n = 18, 1.22%) were the top 3 authors in this field in terms of productivity, and Baselga J showed the highest h-index. Notably, Baselga J had the highest link strength with other authors (total link strength = 8,004). In addition, his 2012 article was the most cited paper, with a total of 2,018 citations, which demonstrated that treatment with the mTOR inhibitor everolimus in combination with an aromatase inhibitor will improve progression-free survival in patients with hormone receptor-positive advanced breast cancer who received prior treatment with a nonsteroidal aromatase inhibitor (11).

The keyword analysis revealed that the application of mTOR inhibitors in the treatment of breast cancer was initially the focus of research in this field. In recent years, the focus of research has shifted to the mechanism of action of the mTOR signaling pathway in the occurrence and development of breast cancer. The keyword analysis and keyword plus analysis revealed resistance to mTOR inhibitors in breast cancer treatment as an emerging research area. The adverse reactions and avoidance measures during the treatment of mTOR inhibitors will be the focus of future research in this field. The high frequency of the keyword “apoptosis” suggests that it is of research value to induce apoptosis through the mTOR signaling pathway for the treatment of breast cancer. Moreover, the emerging keyword “triple negative breast cancer” deserves attention. Triple-negative breast cancer (TNBC) is a highly malignant and aggressive subtype with a poor prognosis and high recurrence rate (26–28). TNBC lacks the expression of progesterone receptor (PR), estrogen receptor (ER) and human epidermal growth factor receptor 2 (HER2) (26, 27, 29, 30). At present, there is no completely effective targeted therapy for TNBC (27, 30). Studies have shown that high expression of mTOR protein is associated with poor prognosis in TNBC patients (27). The mTOR signaling pathway an important and active pathway involved in the occurrence and development of TNBC, which may be a potential target for molecular treatment of TNBC (26, 30). Previous studies have demonstrated that the mTOR inhibitors rapamycin and CCI-779 can inhibit brain metastasis of TNBC at moderate concentration levels (28). In recent years, multiple studies have focused on developing drugs to inhibit the mTOR signaling pathway and treat TNBC (26, 29). In addition to efficacy and toxicity, drug resistance should also be considered when developing drugs targeting the mTOR signaling pathway (27, 29, 31).

In the future, additional effort should be devoted to elucidating the molecular mechanisms of mTOR signaling and resistance in breast cancer. This could provide novel insights for the development of more effective targeted therapies. The increase in the number of articles on triple-negative breast cancer and its relationship with mTOR signaling also represents an emerging area of research. Drugs targeting the mTOR pathway may become a promising treatment option for triple-negative breast cancer. However, issues regarding drug efficacy, safety, and resistance still need to be further investigated and addressed.

Nevertheless, the limitations of the current study should be acknowledged. First, the results retrieved from different databases could not be directly combined due to the limitations of the scientometric analysis software, so the literature search was only performed on Web of Science and not in other databases (e.g., Embase, PubMed, or Scopus). However, Web of Science does not include all journals and focuses more on English-language journals, resulting in an underrepresentation of non-English journals. Still, Web of Science remains the most frequently used literature database in scientometric research. Most bibliometric analysis software can recognize the format of the literature exported from the Web of Science database.

This study represents the initial comprehensive bibliometric analysis of mTOR and breast cancer research from multiple perspectives including countries, institutions, journals, articles, and keywords using Bibliometrix and VOSviewer to assess the current state of research and the evolution of research hotspots in the field. Research in this field is mainly focused on the following aspects: the mechanism of mTOR signaling pathway in breast cancer development, the application and optimization of mTOR inhibitors in breast cancer treatment, and the role of mTOR signaling pathway in breast cancer drug resistance. All these aspects are important elements of breast cancer research, which help to reveal the pathogenesis of breast cancer and improve the treatment outcome.

Meanwhile, drug resistance is a hot topic worthy of in-depth study. The mTOR signaling pathway plays an essential regulatory role in the development of breast cancer, which is also influenced by other signaling pathways or factors, such as PI3K/AKT, AMPK, ER, HER2, IGF-1R, etc. Furthermore, mTOR inhibitors can inhibit the proliferation, differentiation, and self-renewal of breast cancer stem cells, thus eliminating the root cause of breast cancer. However, mTOR inhibitors may also induce breast cancer to evade drug effects (10, 32). The mechanisms of drug resistance include feedback activation of the mTOR signaling pathway, cross-regulation of the mTOR signaling pathway, and escape mechanisms outside the mTOR signaling pathway. Therefore, effectively removing breast cancer stem cells and preventing drug resistance development remains a challenge.

This study offers potential research directions to researchers in related fields.

## 5 Conclusion

The role of mTOR in the development and treatment of breast cancer has been acknowledged around the world. In recent years, many researchers have initiated investigations into approaches aimed at circumventing the adverse outcomes of mTOR inhibitors during the treatment of breast cancer. This scientometric study analyzes information on articles related to the role of mTOR in breast cancer development and treatment during 2012-2022, revealings the authors, institutions, journals, and countries that have contributed to this research area, and providing information on the findings of landmark articles in this research area, as well as research hotspots and research directions. Over the past few decades, there has been a growing trend of research that has centered on the application of mTOR inhibitors in the treatment of breast cancer the mechanism of mTOR signaling pathway in the development of breast cancer and the side effects of mTOR inhibitors in the treatment process. This study provides guidance for the future development of targeted drugs. Emerging topics such as “cdk4”, “triple negative breast cancer”, “esr1 mutations”, and “multicenter” need further research. Preventing drug resistance against mTOR inhibitors is also a topic worth exploring. Considering the heavy treatment burden of cancer patients, the research on mTOR will continue to rise, which will result in progress in clinical treatment modalities.

## Permission to reuse and copyright

Figures, tables, and images will be published under a Creative Commons CC-BY licence and permission must be obtained for use of copyrighted material from other sources (including re-published/adapted/modified/partial figures and images from the internet). It is the responsibility of the authors to acquire the licenses, to follow any citation instructions requested by third-party rights holders, and cover any supplementary charges.

## Data availability statement

The raw data supporting the conclusions of this article will be made available by the authors, without undue reservation.

## Author contributions

JW provided guidance on the use of these analytical applications and revising manuscripts. XZ extracted the dataset from the Web of Science, performed the statistical analysis, and was a major contributor to writing the manuscript. QY, HT, LC, DZ, ZJ, and JC were involved in the interpretation of the study findings. YC and ZL provide financial support. All authors read and gave final approval for the submitted versions. All authors contributed to the article and approved the submitted version.
