# Supplementary material for: A scientometric analysis of research trends on targeting mTOR in breast cancer from 2012 to 2022
Source: Front Oncol. 2023 Aug 10;13:1167154. doi: 10.3389/fonc.2023.1167154 (PMC10448818; doi:10.3389/fonc.2023.1167154)
Supplement: Supplementary file 1 [file DataSheet_1.docx]

Supplementary Material

# Supplementary Figures


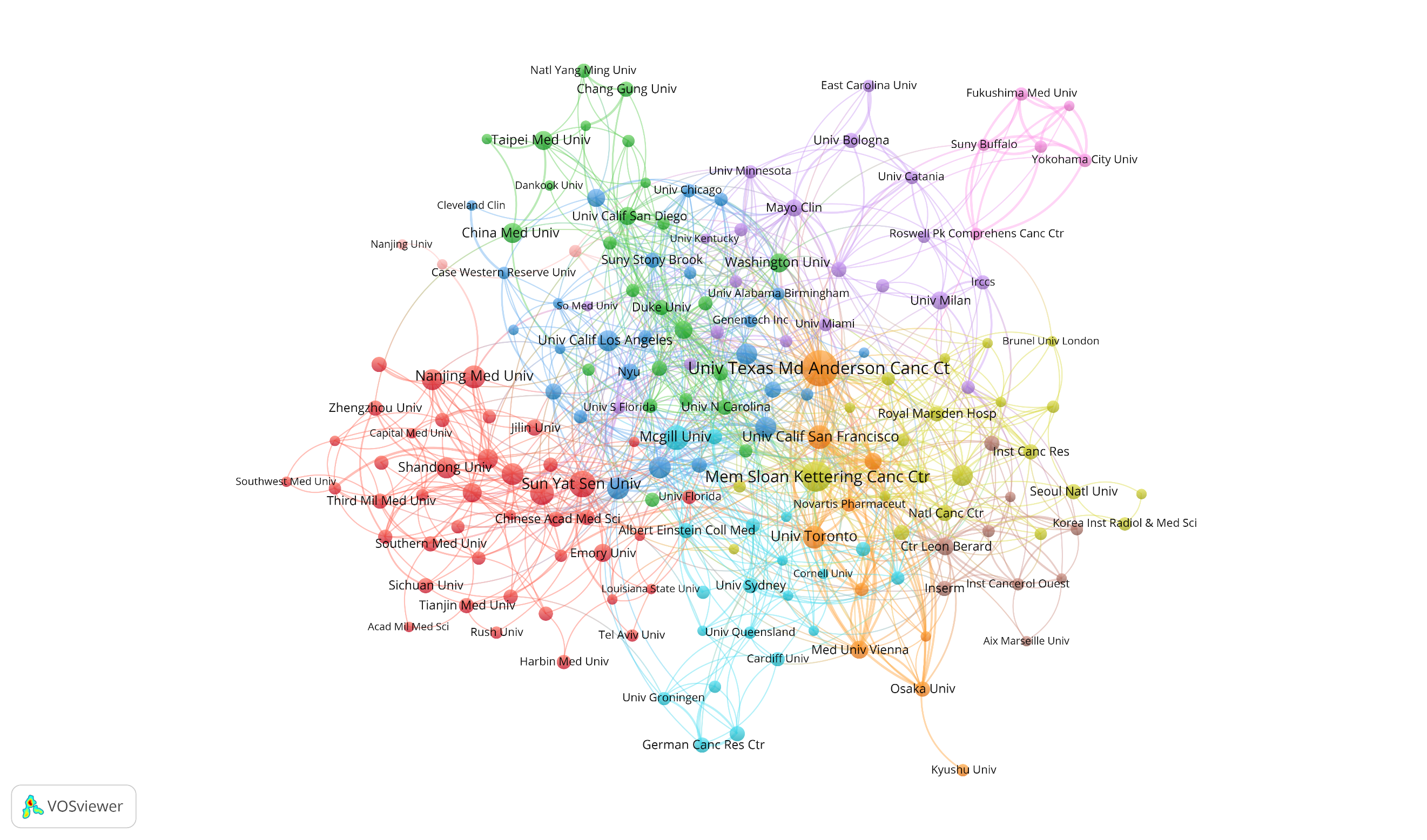


**Supplementary Figure 1.** Network map of co-authorship between organizations in this field.
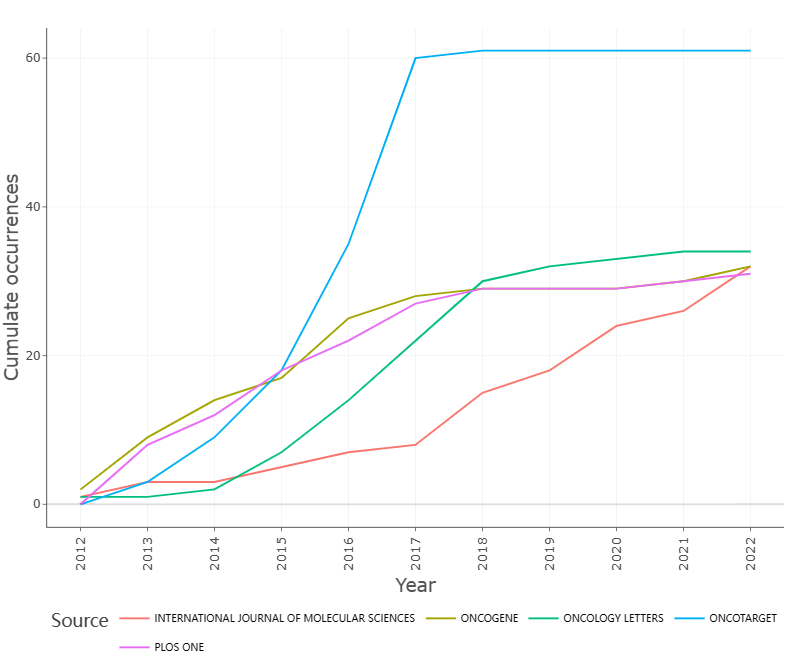


**Supplementary Figure 2.** Source Dynamics Per Year.


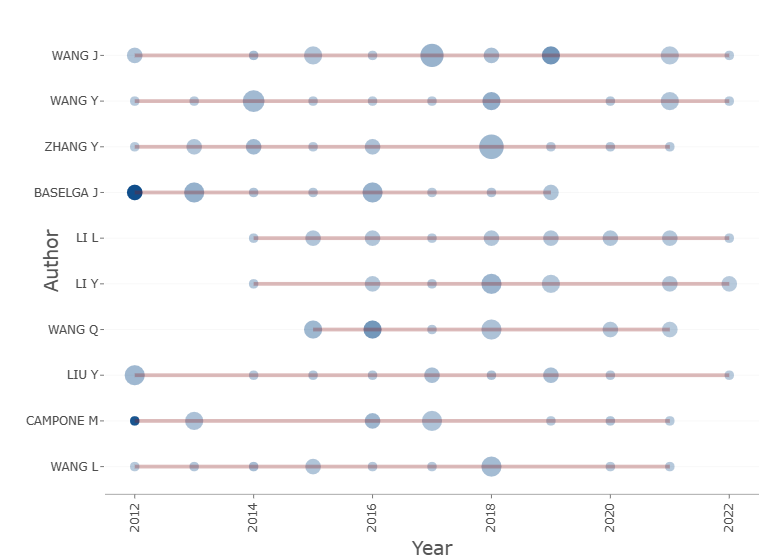


**Supplementary Figure 3.** Authors' Production over Time.


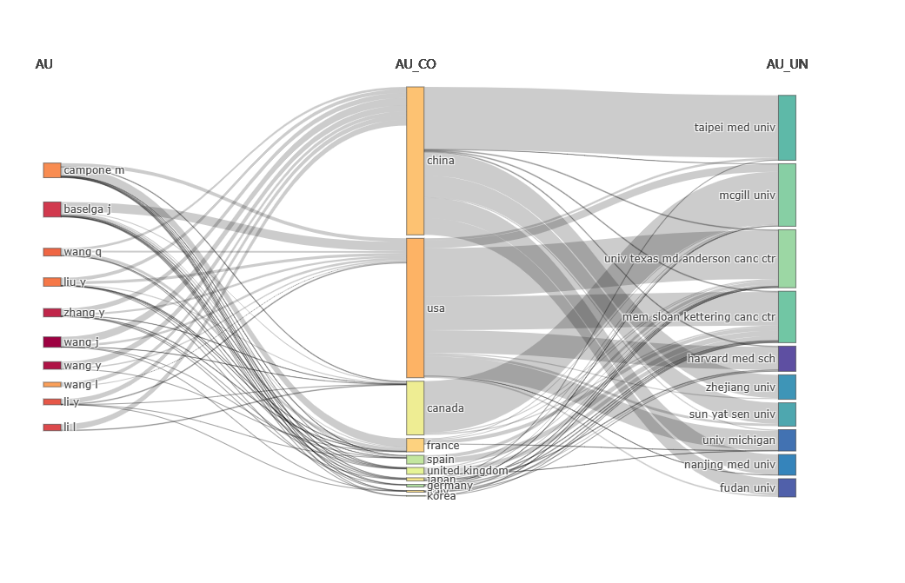


**Supplementary Figure 4.** Three-Field Plot.


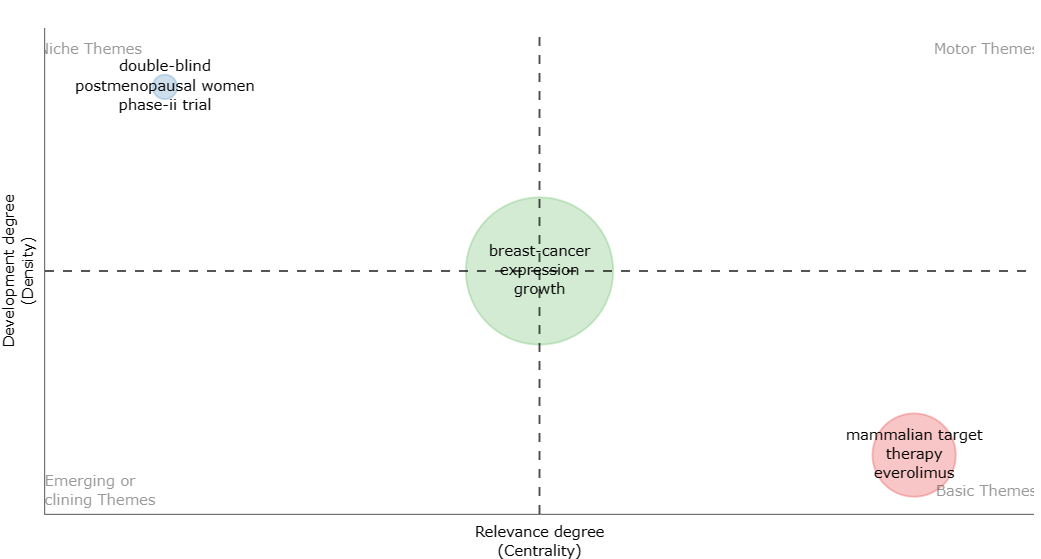


**Supplementary Figure 5.** Thematic Map using Keyword Plus analysis.
